# Supplementary material for: Metal microdrive and head cap system for silicon probe recovery in freely moving rodent
Source: eLife. 2021 May 19;10:e65859. doi: 10.7554/eLife.65859 (PMC8177890; doi:10.7554/eLife.65859)
Supplement: Supplementary file 1. — All animals were implanted with either mouse (M) or rat (R) cap system. The cap system was first tested in a mouse with wire electrodes (M_01) and in a rat without electrophysiology (R_01). After the evaluation of the cap system, we have implanted silicon probes attached to recoverable, plastic microdrives (Plastic recov refers to this microdrive) or flexible probes cemented in place and tested the cap system with electrophysiology in freely moving mice (M_02 – M_04) and in freely moving rats (R_02 – R_06). We used silicon probes from NeuroNexus (Buzsaki-32 NN) and Cambridge Neurotech (ASSY-156 E1 CN). Finally, we evaluated the recoverable, metal microdrive using a Diagnostic Biochips probe (128–5 DB) in a freely moving mouse (M_05) and a Neuropixels probe in a freely moving rat (R_09). All attempted probe recovery was successful except in R_02A (two shanks broke during the acute recordings). The same ASSY-156 E1 (CN) probe was used in R_03 and R_04, but after successful recovery from R_04 the impedance of the contact sites were too high to reimplant this device. A new ASSY-156 E1 probe was used in R_05. [file elife-65859-supp1.docx]

| Animal ID | Strain | Weight (g) | Cap | Drive system | Recording electrode | | Brain region | Acute chronic | Length of recording (days) | Recovery success | Behavior |
| --- | --- | --- | --- | --- | --- | --- | --- | --- | --- | --- | --- |
| M_01^1^ | C57BL/6 | 29 | M | Plastic recov | Tungsten triplet | | Bilateral hippocampus | chronic | 60 | Yes | Homecage |
| M_02 | DBA/2J | 39 | M | NA | 32-ch flexible | | Hippocampus | Chronic | 27 | NA | Homecage |
| M_03 | DBA/2J | 36 | M | Plastic recov | Buzsaki-32 (NN) | | Anterior cingulate ctx | Chronic | 17 | Yes | Homecage open field |
| M_04 | DBA/2J | 37 | M | NA | 32-ch flexible | | Hippocampus | Chronic | 14 | NA | Homecage |
| M_05 | DBA/2J | 38 | M | Metal recov | 128-5 (DB) | | Anterior cingulate ctx | Chronic | 12 | Yes | Homecage open field |
| R_01^2^ | Long Evans | 320 | R | NA | NA | | NA | Chronic | 90 | NA | NA |
| R_02 | Long Evans | 350 | R | NA | 64-ch flexible | | Hippocampus | Chronic | 76 | NA | Homecage linear maze |
| R_03 | Long Evans | 320 | R | Plastic recov | ASSY-156 E1 (CN) | | Hippocampus | Chronic | 16 | Yes | Homecage |
| R_04 | Long Evans | 300 | R | Plastic recov | ASSY-156 E1 (CN) | | Hippocampus | Chronic | 15 | Yes | Homecage |
| R_05 | Long Evans | 340 | R | Plastic recov | ASSY-156 E1 (CN) | | Hippocampus | Chronic | 10 | Yes | Homecage |
| R_06 | Long Evans | 385 | R | NA | 64-ch flexible | | Hippocampus | Chronic | 20 | NA | Nose poke |
| R_07 | Long Evans | 420 | R | Plastic recov | Neuropixels | | Hippocampus | Chronic | 4 | Yes | Homecage |
| R_08 | Long Evans | 330 | R | Plastic recov | Neuropixels from R07 | | Hippocampus | Chronic | Ongoing | NA | Homecage |
| R_09 | Long Evans | 345 | R | Metal recov | Neuropixels | | Hippocampus | Chronic | Ongoing | NA | Homecage linear maze |
| R_01A | Long Evans | 430 | NA | Metal recov | 128-5 (DB) from M05 | Hippocampus | | Acute | NA | NA | NA |
| R_02A | Long Evans | 430 | NA | Metal recov | 128-5 (DB) from R_01A | | Hippocampus | Acute | NA | 2 shank broke | NA |
| M_08 | DBA/2J | 34 | M | Metal recov | 128-5 (DB) from R02_A | | Hippocampus | Chronic | Ongoing | NA | Homecage |

**Supplementary File 1. Summary of experiments using recoverable microdrive and cap system in rodents.** All animals were implanted with either mouse (M) or rat (R) cap system. The cap system was first tested in a mouse with wire electrodes (M_01) and in a rat without electrophysiology (R_01). After the evaluation of the cap system, we have implanted silicon probes attached to recoverable, plastic microdrives (Plastic recov refers to this microdrive) or flexible probes cemented in place and tested the cap system with electrophysiology in freely moving mice (M_02 – M_04) and in freely moving rats (R_02 – R_06). We used silicon probes from NeuroNexus (Buzsaki-32 NN) and Cambridge Neurotech (ASSY-156 E1 CN). Finally, we evaluated the recoverable, metal microdrive using a Diagnostic Biochips probe (128-5 DB) in a freely moving mouse (M_05) and a Neuropixels probe in a freely moving rat (R_09). All attempted probe recovery was successful except in R_02A (2 shanks broke during the acute recordings). The same ASSY-156 E1 (CN) probe was used in R_03 and R_04, but after successful recovery from R_04 the impedance of the contact sites were too high to reimplant this device. A new ASSY-156 E1 probe was used in R_05.
